# The TGF-β/NADPH Oxidases Axis in the Regulation of Liver Cell Biology in Health and Disease

**DOI:** 10.3390/cells10092312

**Published:** 2021-09-03

**Authors:** Macarena Herranz-Itúrbide, Irene Peñuelas-Haro, Rut Espinosa-Sotelo, Esther Bertran, Isabel Fabregat

**Affiliations:** 1 TGF-β and Cancer Group, Oncobell Program, Bellvitge Biomedical Research Institute (IDIBELL), L’Hospitalet de Llobregat, 08908 Barcelona, Spain; mherranz@idibell.cat (M.H.-I.); ipenuelas@idibell.cat (I.P.-H.); respinosa@idibell.cat (R.E.-S.); ebertran@idibell.cat (E.B.); 2 Oncology Program, CIBEREHD, National Biomedical Research Institute on Liver and Gastrointestinal Diseases, Instituto de Salud Carlos III, 28029 Madrid, Spain; 3 Department of Physiological Sciences, Faculty of Medicine and Health Sciences, University of Barcelona, 08907 Barcelona, Spain

**Keywords:** NOX, ROS, TGF-beta, liver regeneration, liver fibrosis, hepatocellular carcinoma, NOX inhibitors

## Abstract

The Transforming Growth Factor-beta (TGF-β) pathway plays essential roles in liver development and homeostasis and become a relevant factor involved in different liver pathologies, particularly fibrosis and cancer. The family of NADPH oxidases (NOXs) has emerged in recent years as targets of the TGF-β pathway mediating many of its effects on hepatocytes, stellate cells and macrophages. This review focuses on how the axis TGF-β/NOXs may regulate the biology of different liver cells and how this influences physiological situations, such as liver regeneration, and pathological circumstances, such as liver fibrosis and cancer. Finally, we discuss whether NOX inhibitors may be considered as potential therapeutic tools in liver diseases.

## 1. Introduction

The Transforming Growth Factor-beta (TGF-β) family of polypeptides plays important roles in the regulation of embryogenesis and adult tissue homeostasis, and it is also implicated in pathophysiological mechanisms that are the basis of several diseases, including fibrosis and cancer [1,2]. The nicotinamide adenine dinucleotide phosphate hydrogen (NADPH) oxidase (NOX) family produces reactive oxygen species (ROS) that play relevant roles in signal transduction pathways [3]. Recent evidence indicates that NOXs may mediate many of the TGF-β effects and, in the opposite way, NOXs could regulate TGF-β activity [4].

## 2. NADPH Oxidase Family Members

In most mammals, seven isoforms of NOX have been described: NOX1–5 and two dual oxidases (DUOX1–2). They use NADPH as an electron donor to reduce oxygen to superoxide anion (O_2_^−^), which is then converted to hydrogen peroxide (H_2_O_2_) either spontaneously or by enzymatic action. The first NOX identified, and the most studied isoform, is NOX2 [5]. Due to its high level of expression in neutrophils and macrophages, NOX2 is known as “phagocyte NADPH oxidase”. Increasingly sensitive detection methods developed during the 1990s made it clear that O_2_^−^ and H_2_O_2_ production was not only restricted to phagocytes, but other cells/tissues also generated them [6]. Since then, the expression of other mammalian NOX homologues has been found in various cell types and tissues, playing roles in maintaining their normal physiology and activity. Dysregulation in NOX expression or activation leads to several pathological consequences, such as cardiovascular diseases, cancer, diabetes, and neurodegenerative diseases [7].

### 2.1. Structural Properties

**Figure 1:**
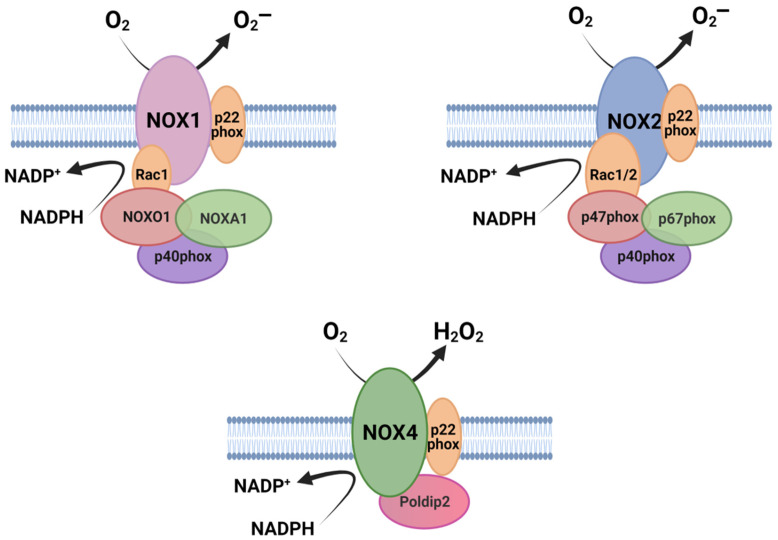
Schematic illustration of the molecular organization of NOX isoforms and their associated subunits. The catalytic core of NOXs is formed by six transmembrane domains (here shown simplified). Figure was created with BioRender.com (accessed on 19 July 2021).

In accordance with their preserved function, NOXs share some common structural properties: (1) the catalytic core of NOX enzymes contains six (seven for DUOX1–2) transmembrane α-helical domains; (2) they present a NADPH-binding site and a flavine adenine dinucleotide (FAD)-binding site at the cytosolic C- terminus; and (3) they contain four highly conserved heme-binding histidines, two in the third and two in the fifth transmembrane domain. Differences between NOX family members are mainly found in their NH2-terminal structure, regulatory proteins and subunits [8,9]. Some members of the family require, for their activation, some cytosolic subunits, which in an inactive state remain dispersed in the cytosol but, upon activation, they assemble with transmembrane subunits to form an active enzyme component [7]. In the case of NOX1, NOX2, and NOX3, the complex is initially formed by the NOX subunit and p22phox, which functions as a maturation factor required for glycosylation, stabilization, and localization of the NOX subunit [10,11,12] (Figure 1). Their activation depends on the small GTPase Rac1 or 2 [13,14] and the recruitment of cytosolic proteins. NOX2 binds p67phox, p47phox [15], and p40phox [16]. NOX1 and NOX3 bind a homolog of p67phox NOXA1 (Nox activator 1) and an homolog of p47phox: NOXO1 (Nox organizer 1) [17,18,19], as well as p40phox. NOXA1 and p67phox are activators that require NOXO1 and p47phox as organizers. As a result of activation, NOX1–3 generate O_2_¯ as a prime product (Figure 1) [20,21,22]. In the case of NOX5, DUOX1, and DUOX2, which are the calcium-activated NOX, they are independent of cytosolic factors but instead have EF-hands for Ca^2+^ sensing. While NOX5 mainly produces O_2_¯ [23,24], DUOX 1–2 produce both O_2_¯ and H_2_O_2_ [25,26]. Finally, NOX4, in contrast to other NOX proteins, produces large amounts of H_2_O_2_, and GTPases Rac are not required for this activity. NOX4 associates with the protein p22phox on internal membranes, where ROS generation occurs [27] (Figure 1). Polymerase-δ-interacting protein 2 (Poldip2) also binds to the complex, promoting NOX4 activity [28,29]. NOX4 is the only NOX constitutively active [27] and it is able to produce H_2_O_2_ through the E-loop, which contains a highly conserved histidine that serves as a source for protons to accelerate spontaneous dismutation of superoxide [30].

NOX isoforms are distinctively expressed in different liver cell types, such as hepatocytes, hepatic stellate cells (HSC), Kupffer cells (KCs), endothelial cells (ECs), and infiltrate leukocytes. Hepatocytes express mRNAs for NOX1, NOX2, NOX4, DUOX1, and DUOX2; KCs, which are resident liver macrophages, express NOX2; in HSCs, NOX1, NOX2, and NOX4 are expressed; and in ECs, NOX1, NOX2, and NOX4 are expressed (Table 1). Nevertheless, considering that NOX1, NOX2, and NOX4 have been widely studied in liver physiology and pathology, in this review we will focus on these three members of the family.

### 2.2. Subcellular Localization of NOXs

Although an excess of ROS is toxic, physiological concentrations of ROS may function as signaling molecules to mediate various responses, including cell migration, growth, and apoptosis. Given that ROS are diffusible and short-lived, producing ROS at the precise subcellular compartment is essential for stimulation of a specific redox signaling pathway, and distinct subcellular localization would explain differences in functions [31]. Nevertheless, the lack of freely available, well-characterized, sensitive antibodies of NOXs for investigative purposes has limited progress of the evaluation of the endogenous expression, localization, and tissue distribution [32]. Furthermore, reported differences may vary depending on the cell type or the overexpression system. In summary, all these obstacles can lead to conflicting reports about the localization of NOX family members.

**Table 1:** Cellular distribution of NOXs in the liver.

Cell Type	Type of NOX	References
Hepatocyte	NOX1, NOX2, NOX4, DUOX1, DUOX2	[33]
HSC *	NOX1, NOX2, NOX4	[33,34]
EC *	NOX1, NOX2, NOX4, DUOX2	[33,34,35,36]
KC *	NOX2	[34,37]
Liver Infiltrating Leukocyte	NOX1, NOX2	[34,38]

* Hepatic Stellate Cell, HSC; Endothelial Cell, EC; Kupffer Cell, KC.

#### 2.2.1. NOX1 and NOX2

NOX1 and NOX2 are classically NOX associated with cell plasma membrane. NOX1 has been seen to co-localize with caveolin, a scaffolding protein associated with caveolae, in punctuate patches of the surface, in Vascular Smooth Muscle Cells (VSMCs) [39,40]. Concomitantly, in hepatocytes, NOX1 activation requires the phosphorylation of sarcoma kinase (Src) by TGF-β, which needs Caveolin 1 and lipid raft domains. NOX1, together with Src, mediate the activation of the tumor necrosis factor (TNF)-α-converting enzyme/a disintegrin and metalloproteinase 17 (TACE/ADAM17), and therefore increases the shedding of different growth factors and cytokines, including the Epidermal Growth Factor Receptor (EGFR) ligands [41]. NOX1 and NOX2 produce ROS in endosomes after a hypoxia-reoxygenation injury, leading to c-Src activation and activity after the recruitment of Rac-1 and c-Src [42]. Fas ligand or TNF-α stimulation of endothelial cells promote the translocation of NOX2 to the lamellipodial leading edge, where ROS induce a rearrangement of the cytoskeleton for cell migration [43,44].

Nonetheless, NOX1 has also been localized in intracellular compartments, such as in the nuclei of keratinocytes [45], but the mechanism involved in its translocation remains unknown. From the nucleus, NOX1 may mediate redox signaling of JNK and ERK cascades [45], similar to what an isoform of NOX4 was demonstrated to do [31], as we will mention later. Localization of NOX2 in perinuclear regions and in the nuclear rich fractions have been also reported [45,46]. NOX2 may also co-localize with calreticulin, a marker of endoplasmic reticulum (ER), in endothelial cells [46].

#### 2.2.2. NOX4

NOX4 has been found in ER, where NOX4-generated ROS oxidize, thus inactivating, the phosphotyrosine phosphatase PTP1B of endothelial cells [47]. It was proposed that NOX4 is required to initiate the oxidative signaling on the ER upstream of the Unfolded Protein Response [48]. In fibroblasts, calnexin (an ER protein) was identified as a NOX4-interacting protein, which was required to maintain NOX4 protein levels and activity [49].

NOX4 plays a role in regulating actin cytoskeleton, whose dynamics are important in growing cells, especially at the leading edge. H_2_O_2_ produced by ER-localized NOX4 may be exported from the organelle to the cytoplasm where it can remodel the actin cytoskeleton. Upon a NADPH oxidase activity blockage, the F-actin fibers network transformed into granular F-actin aggregates, leading to a loss of cell mobility [50]. However, NOX4 has also been seen along actin fibers in a fibrillar form, and also co-localizing with p22phox and vinculin, marking the focal adhesions [39,51]. ROS derived from NOX4 activate Src kinases, which are found in the focal adhesions, thus promoting their adhesion-dependent actions [52]. NOX4 would also oxidize Cysteine residues in F-actin, specifically Cys 272 and 374, promoting the maturation of focal adhesions by means of binding the actin and the vinculin [53].

NOX4 has also been found in the mitochondrial membrane in renal and endothelial cells [54], where ATP negatively regulates NOX4 activity. Mitochondrial NOX4 would function as an energetic sensor of the organelle and as a metabolic checkpoint [55]. Moreover, in cardiomyocytes, NOX4 has been described at the Mitochondria-Associated Membranes (MAMs), the contact sites of endoplasmic reticulum and mitochondria. There, it activates a pro-survival stress pathway that avoids cell death by inhibiting the calcium release in MAMs with its spatially contained ROS signaling [56].

NOX4 localization has also been described to be in the nuclei of different cellular types, such as vascular and endothelial cells, fibroblasts, or hepatocytes, where it has been demonstrated to significantly contribute to hepatic nuclear O_2_^−^ [57]. NOX4 has also been reported to be localized within nuclei in human liver samples [58]. Although in murine hepatocytes, NOX4 protein immunolocalized to both the outer and the inner nuclear membrane, as well as in intranuclear inclusions, fluorescent detection of NADPH-dependent nuclear O_2_^−^ predominantly localized to the perinuclear space [57]. A 28-kDa splice variant of NOX4, known as NOX4D, has been described in the nuclei and nucleolus in VSMCs. This isoform, despite lacking the transmembrane domains, is functionally active and modulates the nuclear signal transduction. NOX4D particularly increases the phosphorylated form of ERK1/2 in the nucleus, which translates into an up-regulation of the transcription factor Elk-1 [31] and may activate redox-sensitive transcription factors, such as c-Fos or c-Jun, involved in growth and differentiation, or NF-κβ, implicated in apoptosis and inflammation [59]. The generation of ROS within the nucleus may also have a relevant role in inactivating nuclear phosphatases [60]. Unregulated nuclear ROS can also cause DNA oxidative damage [31] and can shorten the telomere and modify the DNA and chromosomes, triggering oxidative stress-induced senescence [59].

Finally, NOX4 is also known to be localized in the plasma membrane in VSMCs [39]. This localization was also found when bound to p22phox as a complex, where it constitutively produces H_2_O_2_ [61].

**Figure 2:**
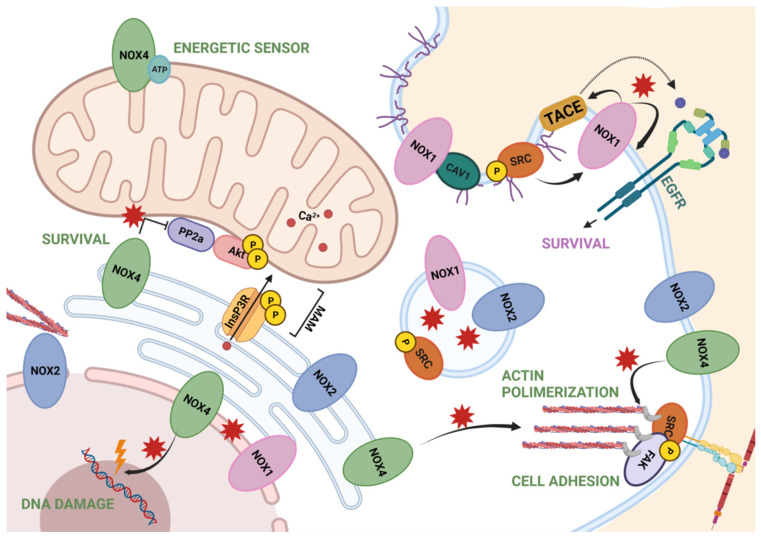
Summary of NOXs localization and NOX-dependent redox signaling through ROS. NOX1 is involved in survival pathways by EGFR and found in endosomes and nuclear membrane. NOX2 is found in the endoplasmic reticulum, endosomes and nuclear membrane, associated with the cytoskeleton. NOX2 is also associated, along with NOX4, to the plasma membrane and near the focal adhesions, regulating cell adhesion. Finally, NOX4 also mediates actin polymerization from the ER, as well as survival signaling when located in MAMs. In the mitochondria, it acts as an energetic sensor, and in the nuclei, is known to produce ROS in the perinuclear and nuclear space. Figure was created with BioRender.com (accessed on 12 July 2021).

A summary of all these data is represented in Figure 2.

## 3. TGF-β in Human Liver Physiology and Pathology

The three isoforms of the TGF-β family (TGF-β1, TGF-β2, and TGF-β3) are synthesized as a pro-form that includes a signal peptide, a large N-terminal region known as latency-associated peptide (LAP), and a short C-terminal sequence that will become the short mature ligand. Once synthesized, it is secreted and anchored to the extracellular matrix (ECM) [62]. TGF-β is synthesized in excess and its activation functions as the rate-limiting step in its bioavailability [63].

**Figure 3:**
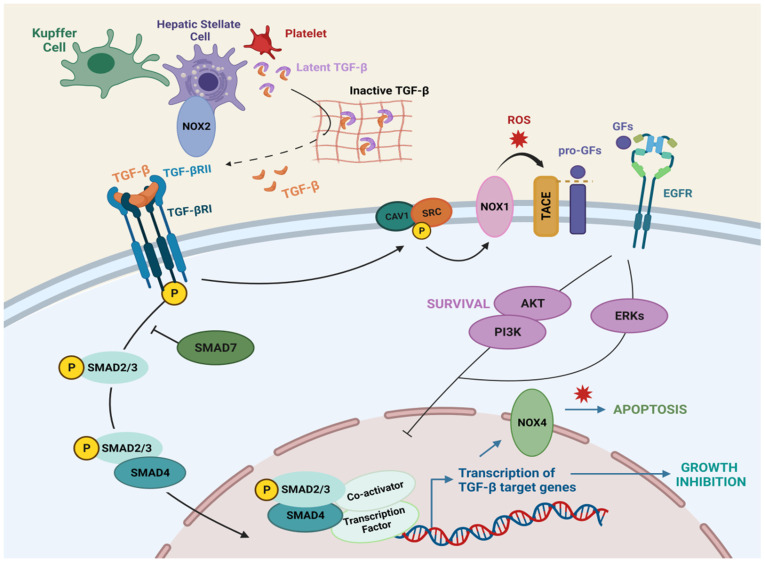
TGF-β signaling in liver cells. TGF-β is synthesized by hepatic stellate cells, kupffer cells and platelets, and anchored to the ECM in a latent form. Once active, TGF-β binds TGFβRII, which recruits TGFβRI, inducing Smad phosphorylation and nuclear translocation, where transcription of target genes occurs. TGF-β-induced shedding of the EGF family of growth factors requires the activation of TACE/ADAM17 in a Caveolin-1/Src/NOX1 dependent manner. Figure was created with BioRender.com (accessed on 20 July 2021).

Upon activation of the dimeric TGF-β from the latent form, TGF-β family members bind to heterotetrameric transmembrane serine/threonine kinases, known as type I and type II receptors. TGF-β binds to homodimeric TGF-βRII, which is constitutively active and phosphorylates and activates homodimeric TGF-βRI. Then, activated TGF-βRI in turn phosphorylates SMAD2 and SMAD3 (receptor-associated SMADs, R-SMADs), which associate with SMAD4 (cooperating-SMAD), forming trimeric complexes that translocate to the nucleus to control the expression of target genes. Moreover, initiation and propagation of TGF-β signaling can be counteracted by the activity of inhibitory-SMADs: SMAD6 and SMAD7 [64,65] (Figure 3). In addition to the canonical SMAD pathway, TGF-β is able to regulate non-SMAD effectors to mediate some of its downstream biological responses, including NF-kB, PI3K/Akt, ERK/JNK/p38 MAPKs, as well as Rho GTPases like RhoA, Cdc42, and Rac1 [64,66].

TGF-β is secreted by several cell types and regulates many aspects of tissue and organ homeostasis. In the liver, TGF-β is mainly secreted by HSCs, KCs, and platelets, but it can be also expressed in regenerating hepatocytes [67] (Figure 3). NOX1 and NOX2 could be mediating the up-regulation of TGF-β expression in HSC cells [34]. In epithelial cells, TGF-β mediates growth inhibition (Figure 3), arresting cells in the G1 phase of the cell cycle, regulating the expression and function of the cyclin-dependent kinase (CDK) inhibitors p21^cip1^ (which inhibits Cyclin E/A-cdk2 complexes) [68] and p15ink4b (which blocks Cyclin D-cdk4/6 complexes) [69,70]. In hepatocytes, TGF-β induces cell cycle arrest and counteracts proliferative signals induced by mitogens, such as Epidermal Growth Factor (EGF) [71,72] or Hepatocyte Growth Factor [73]. TGF-β1 induces p21 through a p53-independent mechanism in rat hepatocytes [74], and cooperation of Smad proteins with Sp1 induces p21 in human hepatoma HepG2 cells [75]. Moreover, TGF-β induces down-regulation of c-Myc [76,77] and inhibition of differentiation (ID) proteins [78], which are transcription factors involved in cellular proliferation and inhibition of differentiation, respectively. In this regard, TGF-β treatment down-regulates c-Myc in hepatocytes [79] and in well-differentiated hepatocellular carcinoma (HCC) cell lines in a Smad dependent manner [80,81].

Moreover, TGF-β also participates in the maintenance of tissue homeostasis through its ability to induce apoptosis (Figure 3). In hepatocytes, TGF-β induces down-regulation of the anti-apoptotic protein Bcl-xL in rat fetal hepatocytes, in TGF-α transgenic mouse hepatocytes and in Hep3B cells [82,83,84], as well as up-regulation of pro-apoptotic proteins Bim and Bax in liver cells [85,86,87]. TGF-β-induced ROS production is required to exert its pro-apoptotic role in hepatocytes [71,83,88]. ROS levels depend on two different mechanisms: (1) induction of NOX4 [89,90] and (2) down-regulation of antioxidant genes and proteins [82,90].

Furthermore, in fetal hepatocytes and liver tumor cells, TGF-β can also induce survival signals through activation of the EGFR pathway and c-Src phosphorylation (Figure 3). EGF counteracts TGF-β-induced cell death effects in hepatocytes via PI3K/Akt pathway [91,92], and blocking the EGFR signaling increases the apoptotic response to TGF-β [93]. As previously mentioned, TGF-β mediates the shedding of EGFR ligands, for which it requires the activation of the metalloprotease TACE/ADAM17 [93,94] in a Caveolin-1/Src/NOX1 dependent manner [41,95]. Importantly, TGF-β-induced activation of NOXs mediates up-regulation of the expression of EGFR ligands, such as TGF-α and HB-EGF, through a NF-kB-dependent mechanism [96]. Furthermore, NOX1 promotes autocrine growth of liver tumor cells through the activation of the EGFR pathway via up-regulation of TGF-α [97].

## 4. Role of the TGF-β/NOX Axis in Liver Regeneration

Liver regeneration (LR) is the response to loss of hepatic tissue [98]. Hepatocytes are the major functional cells of the liver. Indeed, in most cases, the regenerative response is strongly triggered when there is a loss of hepatocytes at a large scale [99]. Chronic loss of hepatocytes is observed in infectious diseases, chronic toxic conditions, ischemia reperfusion injury, or chronic immune attacks and is accompanied by the proliferation of the surviving cells, which may lead to development of neoplasia when occurring in potentially genotoxic environments. The acute loss of hepatocytes, which is less common, can be caused by ingestion of toxins (e.g., intoxication by acetaminophen), trauma, or acute hepatitis. In all these situations, compensatory proliferation, hepatocyte death, and inflammation, which remove dead cells and provide cytokines for repair, proceed in tandem [100]. Nevertheless, when the regenerative process is affected, it may lead to serious liver failure and deterioration in a patient’s general condition [101].

A transient escape of regenerative hepatocytes from TGF-β-induced growth inhibition and apoptosis has been proposed and is achieved through reduction in the expression of TGF-β receptors I and II [102] and the acquisition of survival signals [103], among others. Nevertheless, a mitoinhibitory response to TGF-β is still present in regenerating hepatocytes, since intravenous TGF-β1/2 reversibly inhibits the proliferative response of liver to partial hepatectomy (PH) [104]. Inactivation of the TGF-β pathway by using liver-specific Tgfbr2 knock-out mouse results in an increased proliferative response after PH [105,106], and inhibition of TGF-β signaling also facilitates liver regeneration upon acute dimethylnitrosamine (DMN) [107] or CCl_4_-induced injury [108,109].

Antioxidant treatment improves liver regeneration, indicating that elevated ROS levels could be deleterious during this process [110,111]. Mice lacking the Nrf2 transcription factor, in which the expression of several cytoprotective enzymes was reduced concomitant with higher ROS levels, presented a delayed liver regeneration after PH [112]. Nevertheless, Bai et al. described that H_2_O_2_ promotes adult hepatocytes to transition from quiescence to proliferation in an ERK-dependent pathway, and they also reported an increase in H_2_O_2_ early after two-thirds (2/3) PH [113]. Therefore, ROS levels probably determine the outcome during liver regeneration, and a balanced redox status is necessary for liver regeneration to proceed correctly.

It was initially proposed that ROS derived from a phagocytic-related NOX system are not essential for LR after PH. Knockout mice lacking Cybb (NOX2) presented a normal liver structure and normal TNF-α and IL-6 production after PH, as well as similar levels of hepatocyte DNA replication as those of WT mice [114]. Regarding NOX4, its silencing in untransformed human and mouse hepatocytes increases *in vitro* cell proliferative capacity and *in vivo* analysis in mice revealed that NOX4 expression was down-regulated under physiological proliferative situations of the liver, such as regeneration after PH [115]. Moreover, Nox4-deleted mice presented an accelerated recovery of the liver-to-body weight ratio and increased survival after the surgeries [116]. Hepatocyte proliferation was higher in Nox4-deleted mice, concomitant with increased expression of c-Myc and down-regulation of the TGF-β pathway. Considering that: (1) NOX4 mediates TGF-β-induced apoptosis in hepatocytes [89]; (2) silencing of NOX4 in hepatocytes confer them higher proliferative activity; and (3) NOX4 decreases during liver regeneration [115,116], when hepatocytes are resistant to TGF-β, it could be speculated that some of the suppressor actions of TGF-β during LR could be mediated by NOX4.

## 5. Role of TGF-β/NOX Axis in Liver Fibrosis

One of the most-studied mechanisms of fibrogenesis influenced by ROS is myofibroblast (MFB) activation., whereas high concentrations of ROS induce HSC death, non-toxic levels of ROS stimulate the activation, proliferation, and collagen I production of HSCs [117]. Importantly, activated HSCs have increased ROS-detoxifying capacity compared to quiescent HSCs, which protects them from ROS-induced cell death [118]. Accordingly, MFB activation was shown to be inhibited by antioxidants in the liver [119,120].

**Figure 4:**
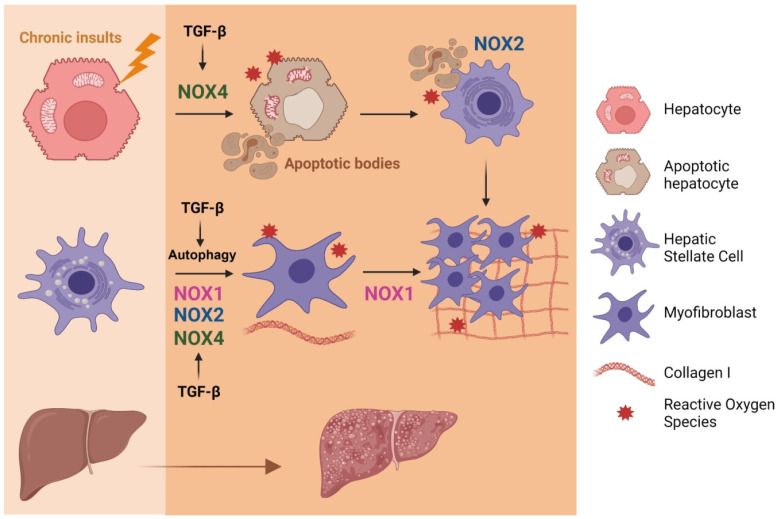
Involvement of the axis TGF-β/NOX in liver fibrosis. Activation of different NOXs by TGF-β contribute to activation of HSC towards MFB, the cells responsive for extracellular matrix proteins deposition. TGF-β-induced NOX4 mediates hepatocyte apoptosis. Apoptotic bodies are phagocytosed by HSCs, a process that requires NOX2, which also contributes to their proliferation and activation towards MFBs. Figure was created with BioRender.com (accessed on 12 July 2021).

TGF-β-induced NOX4 regulates oxidative stress in the liver and levels of TGF-β ligands and NOX4 are increased in fibrotic patients, both virus- and non-alcoholic steatohepatitis (NASH)-related [121,122], as well as in mice with diet-induced steatohepatitis [122]. NOX4 is required for TGF-β-induced activation of HSCs to MFBs in the liver and TGF-β-induced HSC activation is attenuated by NOX4 down-regulation [121] (Figure 4). It is important to highlight that during fibrosis development the highest level of NOX4 expression is found in hepatocytes, followed by MFBs and HSCs [121]. Not only NOX4, but also NOX1 plays important roles in the progression of hepatic fibrosis, promoting proliferation and activation of HSCs [34,123] (Figure 4). The deficiency of NOX1 or NOX4 prevents liver inflammation and fibrosis in mice and NOX1 and NOX4 protein levels are increased in human livers with cirrhosis compared with normal controls [124]. Regarding NOX2, it has been proposed that it mediates profibrogenic effects in both endogenous liver cells and bone marrow-derived cells [34]. Furthermore, recent results indicate that accelerated fibrosis in the aged is modulated by p52Shc/NOX2. A direct activation of NOX2 in hepatocytes by p52Shc binding and activating the p47phox subunit results in redox stress and accelerated fibrosis in the aged [125] (Figure 4). Finally, a role for Nox5 (which is not expressed in mice) has been proposed, mediating the proliferation and activation of human HSC in response to TGF-β, via p38 MAPK [126].

Hepatocyte cell death is a crucial and indirect event during fibrogenesis because apoptotic bodies derived from damaged hepatocytes can transdifferentiate HSCs to MFBs [127]. TGF-β promotes cell death via NOX4 activation in hepatocytes and mouse hepatic oval cells [89,128]. Moreover, NOX4 is also required for FasL or TNF-α-mediated apoptosis [129]. Cytokines through STAT5 also regulate the expression of NOX4 and key pro-apoptotic proteins [130]. Apoptosis and phagocytosis of hepatocytes directly induce HSC activation and initiation of fibrosis (Figure 4). NOX2, the phagocytic NADPH oxidase, plays a key role in this process and in liver fibrogenesis *in vivo*, as it is required for phagocytosis of apoptotic bodies by HSCs [131]. Furthermore, dying hepatocytes release damage-associated molecular patterns that, upon binding to evolutionary conserved pattern recognition receptors, activate cells of the innate immune system to further stimulate inflammatory responses, hence establishing a highly hepatotoxic feedforward cycle of inflammation and cell death [132].

It has been suggested that TGF-β1 mediates epithelial-mesenchymal transition (EMT) in hepatocytes, which may transdifferentiate them to MFBs, contributing to liver fibrosis [133]. There is no evidence for the direct involvement of NOXs in this process [121], but it has been suggested that the EMT process is dependent on the presence of the NLRP3 (NACHT, LRR, and PYD domain-containing protein 3) receptor [134]. The EMT process might happen in two ways: one is dependent on the inflammasome activation, when the IL-1β executes its proinflammatory action by up-regulating the TGF-β pathway; another is independent of the inflammasome activation, being necessary only in the presence of the NLRP3 receptor which enhances R-Smads in the TGF-β/Smad pathway [134].

Finally, autophagy activated by ROS is emerging as a novel mechanism of hepatic fibrosis [135]. TGF-β-mediated ROS production, in a NOX4-dependent pathway, induces autophagy in epithelial cells [136]. During liver fibrosis, TGF-β1 induces autophagy through activation of the ERK and JNK signaling pathways [137], which play essential roles in regulating autophagic processes [138], and autophagy is involved in the activation of HSCs [137]. In fact, using cell culture and/or different animal models, numerous studies have shown that autophagy is enhanced during the fibrogenic process and have provided specific evidence to pinpoint the fundamental role of autophagy in HSCs activation [139]. A recent study indicates that alamandine attenuates liver fibrosis by regulating autophagy induced by NOX4-dependent ROS [140].

## 6. Role of NOXs in HCC

### 6.1. NOXs in HCC

**Figure 5:**
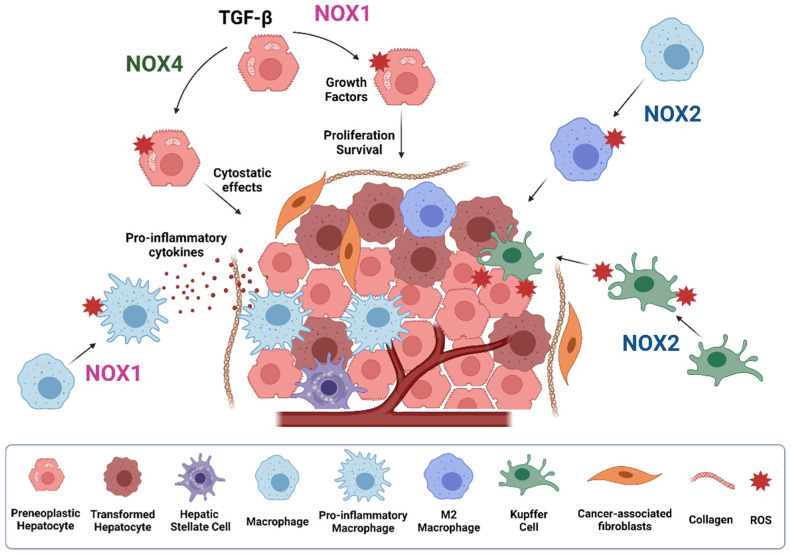
Role of NOXs during HCC development. NOX1 and NOX4 play opposite roles in hepatocytes and liver tumor cells. While NOX4 induces cytostasis, NOX1 mediates proliferative and survival signals. NOX1 also mediates activation of macrophages to pro-inflammatory macrophages, promoting inflammatory cytokines secretion. NOX2 promotes M2 macrophage polarization and superoxide production by kupffer cells, contributing to the promotion of HCC. Figure was created with BioRender.com (accessed on 20 July 2021).

Liver fibrosis can lead to cirrhosis, a condition that highly predisposes a person to develop HCC, which is the most common primary liver cancer that usually occurs under a situation of chronic liver insults [141,142]. The onset and progression of HCC is a multistep process usually involving subsequent mutations in genes that control cellular proliferation and/or apoptosis in hepatocytes that are subjected to continuous inflammatory and regenerative stimuli [143,144]. During malignant transformation and hepatic carcinogenesis, ROS are overwhelmingly produced creating an oxidative microenvironment that may generate different and various types of cellular stress, including DNA damage, ER stress, cell death of damaged hepatocytes, as well as oxidative stress. Members of the NADPH oxidase family, such as NOX1, NOX2, and NOX4, have been clearly linked to produce ROS in the liver [4], which may contribute to HCC development. Indeed, different NOX subunits, including p47phox, p67phox, and Rac1, were found increased in pre-neoplastic and neoplastic lesions from c-Myc, TGF-β, and c-Myc/TGF-β transgenic mice, where HCC developed under a context of high ROS content and decreased antioxidants in hepatocytes [145]. Although the exact role of NOX proteins in hepatocarcinogenesis is still under investigation, different NOXs, produced in different liver cells, may contribute or inhibit liver cancer progression (Figure 5), as we detail below.

Carcinogenesis can be initiated after chronic inflammatory insults. Recent evidence indicates that NOX1 expression in macrophages mediates tumor promoting activity, through activating a ROS/MEK mechanism responsible of inflammatory cytokines production, thereby promoting the survival and proliferation of oncogene-carrying mutant hepatocytes, which ultimately accelerate HCC development [38]. Pharmacological inhibition of NOX1 with GKT771 during HCC progression in mice attenuates the expression of several inflammatory markers, angiogenesis and fibrosis, therefore reducing the pro-tumorigenic environment [146]. Besides NOX1, NOX2-derived ROS are also involved in TLR2-dependent M2 macrophage polarization, supporting tumor growth. In detail, hepatoma-derived HMGB1 stimulates NOX2-derived ROS production via TLR2 to trigger autophagy formation, which leads to lysosomal degradation of NF-kB p65 and hence maintains the M2 macrophage polarization [147]. TLR4 and intestinal microbiota are also key factors in HCC promotion, mediating increased proliferation and preventing apoptosis [148,149]. NOX2 has also been reported to be a relevant mediator of LPS/TLR-4-induced ROS production [145,150]. Indeed, the early effects of alcohol on liver injury involve LPS stimulation of Kupffer cells, which activates NOX2/p47phox, aiding to TNF-α release and subsequent pro-inflammatory environment formation [151]. The generated O_2_^−^ by NOX2 activation during liver inflammation also induces hepatocyte DNA damage, which ultimately may contribute to tumor initiation and promotion, an effect that was attenuated in p47phox-deficient mice [152]. Importantly, an additional study using the same mouse model found that superoxide production from Kupffer cells upon NOX2 activation is more relevant for the promotion rather than for the initiation of hepatocarcinogenesis [153]. In agreement, recent studies suggested that NOX2 expression was significantly induced in HCC patients with hepatic cirrhosis and negatively correlated with advanced tumor stage [58].

A growing body of evidence has focused on the importance of cell metabolic reprogramming during malignant transformation and HCC development to support high proliferation rates. In this line, NOX1 has been found to support the remodeling of cellular metabolism to allow available resources to be redirected towards biosynthesis of macromolecules necessary for cell growth [154,155]. In this regard, NOX1 is involved in glucose and glutamine catabolism, as well as in lipid, protein, and nucleotide anabolism, thereby contributing to HCC [154]. Besides the role of NOX1 in HCC development and cell proliferation, NOX1 has also been reported to be involved in latter stages of HCC, such as in the regulation of cell migration. Mechanistically, NOX1 is negatively regulated by SHMT1 (Serine hydroxymethyltransferase 1), a key enzyme involved in the regulation of one carbon metabolism. Indeed, SHMT1 acts as tumor suppressor as inhibits HCC metastasis, EMT and MMP2 by repressing NOX1-derived ROS production [156].

Despite NOX1 and NOX2 seeming to be inducers/promoters of HCC, recent findings postulate NOX4 as a tumor suppressor. Indeed, NOX1 and NOX4 may have an opposite prognosis in HCC patients after a hepatectomy, those patients with high NOX1 or low NOX4 expression being associated with worse recurrence-free and overall survival rates. [157]. An additional study has demonstrated that higher NOX4 mRNA expression levels in HCC patients are significantly associated with prolonged overall survival, whereas increased NOX1/NOX2 expression is significantly correlated with a poor overall survival, therefore indicating that while NOX4 behaves as a tumor suppressor in HCC, NOX1 and NOX2 may act as tumor promoters [155]. Importantly, NOX4 localization seems to be crucial for its effects as tumor suppressor. Increased nuclear and decreased cytoplasmic NOX4 expression was found in HCC cells, as compared to non-tumoral hepatocytes, correlating with a poorer overall survival [58]. Nuclear NOX4 localization has also been found in chronic hepatitis C virus (HCV) infection, increasing the probability of DNA damage in the liver, a common feature in HCC [158]. In support to its potential tumor suppressor role, NOX4 inhibits liver cell proliferation either under physiological conditions or during tumorigenesis. NOX4 expression is down-regulated in regeneration after PH, as well as during diethyl-nitrosamine (DEN)-induced hepatocarcinogenesis [115]. In fact, NOX4 silencing increased cell proliferation, which is correlated with a higher percentage of cells in S/G2/M phases of the cell cycle, the down-regulation of p21^cip1^, an increase in cyclin D1, and the nuclear localization of β-catenin. Accordingly, silencing NOX4 conferred an advantage to the human HCC cells, resulting in the earlier onset of tumor formation and an increase in tumor size in xenograft mice [115]. In addition to differences in cell growth, NOX4 down-regulation is also correlated with increased migratory and invasive capabilities of HCC cells. NOX4 appears necessary to maintain parenchymal structures, as its attenuation promotes a decrease in cell-to-cell contacts and cell-to-matrix adhesion, correlating with an increase in actomyosin contractility and amoeboid invasion. Therefore, the loss of NOX4 favors an epithelial to amoeboid transition, contributing to tumor aggressiveness [159].

### 6.2. TGF-β, NOXs and HCC

Accumulating data indicate the importance of NOX1 and NOX4 in liver tumor cells downstream TGF-β. As previously mentioned in hepatocytes, NOX4 mediates the pro-apoptotic effects of TGF-β in HCC cells, involving STAT5 and *PUMA*, *BIM*, and *BMF* gene up-regulation [89,130,160]. Furthermore, it has been reported that NOX4 is responsible for TGF-β-induced senescence in HCC cells [80]. Overactivation of survival signals, such as the MEK/ERK pathway, confers resistance to TGF-β-induced cell death by impairing the up-regulation of NOX4 [160]. On the contrary, NOX1 is considered as a survival signal in liver tumor cells as it has been found to play relevant roles in mediating pro-tumorigenic TGF-β effects. NOX1 mediates autocrine growth and the survival of liver tumor cells, as well as the anti-apoptotic signals induced by TGF-β through the transactivation of the EGFR pathway and TGF-β expression, involving p38 MAPK and AKT activation [97]. Indeed, NOX1 pharmacological inhibition with VAS2870 impairs cell growth and enhances TGF-β- induced apoptosis [161]. Importantly, the inhibition of the EGFR pathway enhances TGF-β-induced apoptosis [162] and correlates with higher levels of the NADPH oxidase NOX4 [163]. The EGFR transactivation mediated by NOX1 results in the activation of the NF-κB pathway which, in turn, up-regulates EGFR ligands together with TACE/ADAM17 metalloprotease activation, which are responsible for the shedding of these ligands [93,164]. Experiments of loss or gain of function in HCC cells have revealed an essential role for clathrin in the activation of the EGFR by TGF-β, which impairs its apoptotic activity by inhibiting the expression of its pro-apoptotic target NOX4 [165]. Overall, NOX1 and NOX4 exert opposite roles in the control of liver growth and apoptosis and their balance may dictate cell fate [162]. Indeed, increased activation of tyrosine kinase activities or decreased activity of protein phosphatases, such as PTP1B, increases the NOX1/NOX4 ratio, which is responsible of NF-κB nuclear translocation that subsequently mediates resistance to TGF-β suppressor effects [166].

## 7. NOX Inhibitors as Therapeutic Tools in Liver Diseases

Although inhibiting TGF-β may be considered a therapeutic tool in liver diseases [167,168], the discovery of NOXs as TGF-β targets mediating its detrimental effects in liver inflammation and fibrosis pushed to the challenge of using NOX inhibitors as a more specific and adequate way to attack fibrotic pathologies [169]. However, the development of specific inhibitors of NOX enzymes has proved difficult due to the lack of a crystal structure for all NOX enzymes, as well as the complexity of their regulatory interactions. Indeed, the specificity of NOX inhibitors remains controversial. Different widely used compounds, such as diphenyleneiodonium (DPI) or VAS2870 (Vasopharm GmbH), may inhibit all NOXs [170]. The recent development of small-molecule, more specific NOX inhibitors in different academic institutions and private companies led to the use of these new compounds in preclinical assays and, in some cases, clinical trials. Related to liver pathologies, GKT137831 (Genkyotex), which was proposed as a dual NOX4/NOX1, decreased both the apparition of fibrogenic markers and hepatocyte apoptosis *in vivo* upon bile duct ligation and CCl_4_ treatment [122,129,171]. In the same line of evidence, therapeutic blocking NOX1/4 with GKT137831 ameliorated cholestatic fibrosis in Mdr2−/− mice [172]. Furthermore, GKT137831 treatment also prevented liver inflammation after CCl_4_ injection [124] and protected from GSH depletion, caspase-3 cleavage and hepatocyte death in acetaminophen-induced liver injury [173]. In a phase 2 clinical trial, GKT137831 was found to be safe and well tolerated (https://clinicaltrials.gov/ct2/show/NCT02010242, accessed on 7 July 2021). Nevertheless, recent evidence suggests that GKT137831 is not active only on NOX enzymes, but rather has an oxidant scavenging effect [170]. In conclusion, GKT137831, or analogs, might be promising therapeutic tools in chronic liver injury and fibrosis; however, their mechanism of action is not fully explained by NOX inhibition and further studies are needed to better explain its effectiveness.

Due to the evidence that indicates high NOX1 levels in HCC that correlate with a poor prognosis [155,157], as well as the involvement of NOX1 in tumor cell proliferation and migration [156] and macrophage-induced inflammation [38], different studies focused on the use of NOX1 inhibitors in experimental models of hepatocarcinogenesis. DEN-injected wild-type (WT) mice that received a NOX1 inhibitor ML171 [174] developed fewer and smaller hepatic tumor nodules, compared to their vehicle-treated counterparts [38]. In agreement, a recent report indicated that pharmacological inhibition of NOX1 with GKT771 (Genkyotex) during HCC progression in mice attenuate the expression of several inflammatory markers, angiogenesis and fibrosis, therefore reducing the pro-tumorigenic environment [146]. Despite the opposite role of NOX1 and NOX4 in HCC (the first one promoting and the second one suppressing tumor progression), the fact that most of the liver tumor cells express low levels of NOX4 [115] facilitates the use of general NOX inhibitors as promising tools in the treatment of liver cancer. In this sense, VAS2870 has proved to be very effective in inhibiting proliferation and enhancing apoptosis induced by a physiological stimulus, such as TGF-β in HCC cells. Nevertheless, all these studies are in a preliminary phase and further work is necessary to fully conclude that NOXs may be therapeutic targets in liver cancer.

## 8. Conclusions

Taken together the data found in the literature, there is no doubt that the cross-talk between TGF-β and NOXs mediate relevant functions in liver cell biology, both under physiological and pathological conditions. TGF-β-mediated activation of NOX-produced ROS appears to be toxic in non-neoplastic conditions, contributing to hepatocyte cell death and an inflammatory environment, which determines the transition towards a pro-fibrotic chronic disease. The scenario becomes more complicated when considering proliferative situations, such as liver regeneration, which occurs concomitantly to chronic liver diseases, or hepatocarcinogenesis, where TGF-β-mediated NOX activation would play opposite roles, depending on the member of the NOX family and the intracellular localization where ROS are produced. Further studies are necessary to better understand the axis TGF-β/NOX and their relationship with chronic liver diseases, fibrosis, and cancer to validate the relevance of targeting specific NOXs in liver pathologies. Furthermore, the development of specific inhibitors of each NOX isoform is absolutely necessary in a scenario so complex where different NOXs could play opposite functions.
